# Urgent Surgery

**Published:** 1910-09

**Authors:** 


**Urgent Surgery.** By Félix Lejars. Translated from the Sixth French Edition by W. S. Dickie, F.R.C.S. Vol. I. Pp. xiv, 617. Bristol: John Wright & Sons Ltd. 1909.

The translator and publishers of this work are to be congratulated on its material and artistic excellence, and its production is an honour to its author and an ornament to European surgery. There are few authors who can say of a book, "I have lived it before writing it," and the text bears evidence, in its easy, demonstrative style, and in its judicious selection of facts and illustrations, that the author lives what he writes. With the collaboration of artists of the French school, who have produced some of the finest line drawings we have ever seen in surgical literature, and with the addition of some 200 photographs, M. Lejars has produced a monumental surgical volume of an admirable character. We cordially commend the work to our surgical friends, and look forward to the appearance of the next volume.

